# Supplementary material for: Adaptor protein supersaturation drives innate immune signaling and cell fate
Source: eLife. 2026 Mar 24;14:RP107962. doi: 10.7554/eLife.107962 (PMC13012725; doi:10.7554/eLife.107962)
Supplement: Supplementary file 2. — FL (full-length). aMutations were made to inactivate enzymatic activity for cell death effectors. [file elife-107962-supp2.pdf]

		construct analyzed					DAmFRET results
**protein name (alias)**	**species**	**uniprot**	**plasmid**	**subfamily**	**beginning-end (mutation)[a]**	**term fused**	**DAmFRET classification**
AIM2	*H. sapiens*	O14862	rhx4773	PYD	FL	C-term	continuous (low to high)
BCL10	*H. sapiens*	O95999	rhx2138	CARD	FL	C-term	discontinuous
CARD11	*H. sapiens*	Q9BXL7	rhx2230	CARD	FL	C-term	continuous (low to high)
CARD19	*H. sapiens*	Q96LW7	rhx3053	CARD	FL	C-term	continuous (low to high)
CARD9	*H. sapiens*	Q9H257	rhx2137	CARD	FL	C-term	continuous (low to high)
CASP1	*H. sapiens*	P29466	rhx3048	CARD	FL (C285A)	C-term	continuous (low to high)
CASP2	*H. sapiens*	P42575	rhx4777	CARD	FL (C320S)	C-term	continuous (high)
CASP5	*H. sapiens*	P51878	rhx2583	CARD	FL (C135A)	N-term	continuous (low)
CASP8	*H. sapiens*	Q14790	rhx2588	tDED	FL (C360A)	C-term	continuous (low to high)
CASP9	*H. sapiens*	P55211	rhx2640	CARD	FL (C287A)	C-term	continuous (low to high)
CRADD	*H. sapiens*	P78560	rhx1078	CARD, DD	FL	C-term	continuous (low to high)
DEDD2	*H. sapiens*	Q8WXF8	rhx4648	DED	FL	N-term	continuous (low to high)
EDARADD	*H. sapiens*	Q8WWZ3	rhx4770	DD	FL	N-term	continuous (high)
FADD	*H. sapiens*	Q13158	rhx1312	DED, DD	FL	C-term	discontinuous
MALT1	*H. sapiens*	Q9UDY8	rhx2139	DD	FL	C-term	continuous (low)
MAVS	*H. sapiens*	Q7Z434	rhx1379	CARD	1-100	C-term	discontinuous
MAVS	*H. sapiens*	Q7Z434	rhx2387	CARD	FL	N-term	discontinuous
MAVS	*H. sapiens*	Q7Z434	rhx4859	CARD	1-100	N-term	continuous (low to high)
MYD88	*H. sapiens*	Q99836	rhx1311	DD, TIR	FL	C-term	continuous (high)
NLRP3	*H. sapiens*	Q96P20	rhx2238	PYD	FL	C-term	continuous (low to high)
PYCARD (ASC)	*H. sapiens*	Q9ULZ3	rhx0927	PYD, CARD	FL	C-term	discontinuous
RIPK1	*H. sapiens*	Q13546	rhx3250	RHIM, DD	FL	C-term	continuous (high)
TRADD	*H. sapiens*	Q15628	rhx3079	DD	FL	C-term	discontinuous
CASP1	*D. rerio*	Q9I9L7	rhx5587	PYD	2-112	C-term	continuous (low)
CASP1	*D. rerio*	Q9I9L7	rhx5581	PYD	FL (C270S)	C-term	discontinuous
NLRP3	*D. rerio*	A0A8M6YY09	rhx5582	PYD	FL	C-term	continuous (low to high)
NLRP3	*D. rerio*	A0A8M6YY09	rhx5588	PYD	2-97	C-term	discontinuous
PYCARD (ASC)	*D. rerio*	Q9I9N6	rhx4009	PYD, CARD	FL	C-term	discontinuous
PYCARD (ASC)	*D. rerio*	Q9I9N6	rhx5454	PYD	2-111	C-term	discontinuous
PYCARD (ASC)	*D. rerio*	Q9I9N6	rhx5586	CARD	92-203	C-term	discontinuous
CASP8	*A. queenslandica*	A0A1X7VAD9	rhx5585	tDED	FL	C-term	continuous (low to high)
CASP8	*A. queenslandica*	A0A1X7VAD9	rhx5591	tDED	2-233	C-term	discontinuous
FADD	*A. queenslandica*	A0A1X7UHE5	rhx4796	DED,DD	FL	C-term	discontinuous
FADD	*A. queenslandica*	A0A1X7UHE5	rhx5670	DED	2-110	C-term	discontinuous
FADD	*A. queenslandica*	A0A1X7UHE5	rhx5671	DD	102-197	C-term	discontinuous
NACHT domain-containing protein	*A. queenslandica*	A0A1X7U321	rhx5589	DD	2-168	C-term	continuous (high)
NACHT domain-containing protein	*A. queenslandica*	A0A1X7U321	rhx5583	DD	FL	C-term	continuous (low to high)
